# Supplementary material for: A genetic, genomic, and computational resource for exploring neural circuit function
Source: eLife. 2020 Jan 15;9:e50901. doi: 10.7554/eLife.50901 (PMC7034979; doi:10.7554/eLife.50901)
Supplement: Supplementary file 2. — List of key resources and reagents used in this study. [file elife-50901-supp2.docx]

| **Key Resources Table** | | | | | |
| --- | --- | --- | --- | --- | --- |
| **Reagent type (species) or resource** | **Designation** | **Source or reference** | **Identifiers** | **Additional information** |  |
| genetic reagent  (*Drosophila melanogaster*) | C2_d1; SS00778 | [https://doi.org/10.1016/j.neuron.2013.05.024](https://doi.org/10.1016/j.neuron.2013.05.024) | SS00778 | For further details on driver lines see Table S1. Available via <https://www.janelia.org/split-GAL4>. |  |
| genetic reagent  (*D. melanogaster*) | C2.C3_d1; SS00779 | [https://doi.org/10.1016/j.neuron.2013.05.024](https://doi.org/10.1016/j.neuron.2013.05.024) | SS00779 | For further details on driver lines see Table S1. Available via <https://www.janelia.org/split-GAL4>. |  |
| genetic reagent  (*D. melanogaster*) | C3_d1; SS00688 | [https://doi.org/10.1016/j.neuron.2013.05.024](https://doi.org/10.1016/j.neuron.2013.05.024) | SS00688 | For further details on driver lines see Table S1. Available via <https://www.janelia.org/split-GAL4>. |  |
| genetic reagent  (*D. melanogaster*) | CCAP_d1 | Bloomington Drosophila Stock Center (BDSC) | BL-25686 | For further details on driver lines see Table S1. |  |
| genetic reagent  (*D. melanogaster*) | ChAT_d1 | BDSC | BL-60317 | For further details on driver lines see Table S1. |  |
| genetic reagent  (*D. melanogaster*) | Crz_d1 | BDSC | BL-51977 | For further details on driver lines see Table S1. |  |
| genetic reagent  (*D. melanogaster*) | Dm1_d1; SS00309 | This paper | SS00309 | For further details on driver lines see Table S1. Available via <https://www.janelia.org/split-GAL4>. |  |
| genetic reagent  (*D. melanogaster*) | Dm10_d1; SS00327 | This paper | SS00327 | For further details on driver lines see Table S1. Available via <https://www.janelia.org/split-GAL4>. |  |
| genetic reagent  (*D. melanogaster*) | Dm11_d1; SS03159 | This paper | SS03159 | For further details on driver lines see Table S1. Available via <https://www.janelia.org/split-GAL4>. |  |
| genetic reagent  (*D. melanogaster*) | Dm12_d1; SS00384 | This paper | SS00384 | For further details on driver lines see Table S1. Available via <https://www.janelia.org/split-GAL4>. |  |
| genetic reagent  (*D. melanogaster*) | Dm3_d1; SS00974 | This paper | SS00974 | For further details on driver lines see Table S1. Available via <https://www.janelia.org/split-GAL4>. |  |
| genetic reagent  (*D. melanogaster*) | Dm4_d1; SS02361 | This paper | SS02361 | For further details on driver lines see Table S1. Available via <https://www.janelia.org/split-GAL4>. |  |
| genetic reagent  (*D. melanogaster*) | Dm4_d2; SS02592 | This paper | SS02592 | For further details on driver lines see Table S1. Available via <https://www.janelia.org/split-GAL4>. |  |
| genetic reagent  (*D. melanogaster*) | Dm8_d1; SS00323 | This paper | SS00323 | For further details on driver lines see Table S1. Available via <https://www.janelia.org/split-GAL4>. |  |
| genetic reagent  (*D. melanogaster*) | Dm9_d1; SS02427 | This paper | SS02427 | For further details on driver lines see Table S1. Available via <https://www.janelia.org/split-GAL4>. |  |
| genetic reagent  (*D. melanogaster*) | Dsk_d1 | BDSC | BL-51981 | For further details on driver lines see Table S1. |  |
| genetic reagent  (*D. melanogaster*) | Gad1_d1 | BDSC | BL-60324 | For further details on driver lines see Table S1. |  |
| genetic reagent  (*D. melanogaster*) | Glia_Eg_d1; SS55439 | This paper | SS55439 | For further details on driver lines see Table S1. Available via <https://www.janelia.org/split-GAL4>. |  |
| genetic reagent  (*D. melanogaster*) | Glia_Eg_d2; SS55438 | This paper | SS55438 | For further details on driver lines see Table S1. Available via <https://www.janelia.org/split-GAL4>. |  |
| genetic reagent  (*D. melanogaster*) | Glia_Eg_d3; SS08909 | This paper | SS08909 | For further details on driver lines see Table S1. Available via <https://www.janelia.org/split-GAL4>. |  |
| genetic reagent  (*D. melanogaster*) | Glia_Mg_d1; SS55437 | This paper | SS55437 | For further details on driver lines see Table S1. Available via <https://www.janelia.org/split-GAL4>. |  |
| genetic reagent  (*D. melanogaster*) | Glia_Psg_d1; SS55440 | This paper | SS55440 | For further details on driver lines see Table S1. Available via <https://www.janelia.org/split-GAL4>. |  |
| genetic reagent  (*D. melanogaster*) | Glia_Psg_d2; SS55441 | This paper | SS55441 | For further details on driver lines see Table S1. Available via <https://www.janelia.org/split-GAL4>. |  |
| genetic reagent  (*D. melanogaster*) | Ilp2_d1 | BDSC | BL-37516 | For further details on driver lines see Table S1. |  |
| genetic reagent  (*D. melanogaster*) | KC_ab_c_d1; MB594B | <https://doi.org/10.7554/eLife.04577.047> | MB594B | For further details on driver lines see Table S1. Available via <https://www.janelia.org/split-GAL4>. |  |
| genetic reagent  (*D. melanogaster*) | KC_ab_c.p.s_d1; MB008B | <https://doi.org/10.7554/eLife.04577.047> | MB008B | For further details on driver lines see Table S1. Available via <https://www.janelia.org/split-GAL4>. |  |
| genetic reagent  (*D. melanogaster*) | KC_ab_p_d1; MB371B | <https://doi.org/10.7554/eLife.04577.047> | MB371B | For further details on driver lines see Table S1. Available via <https://www.janelia.org/split-GAL4>. |  |
| genetic reagent  (*D. melanogaster*) | KC_ab_s_d1; MB185B | <https://doi.org/10.7554/eLife.04577.047> | MB185B | For further details on driver lines see Table S1. Available via <https://www.janelia.org/split-GAL4>. |  |
| genetic reagent  (*D. melanogaster*) | KC_apbp_ap_d1; MB463B | <https://doi.org/10.7554/eLife.04577.047> | MB463B | For further details on driver lines see Table S1. Available via <https://www.janelia.org/split-GAL4>. |  |
| genetic reagent  (*D. melanogaster*) | KC_apbp_m_d1; MB418B | <https://doi.org/10.7554/eLife.04577.047> | MB418B | For further details on driver lines see Table S1. Available via <https://www.janelia.org/split-GAL4>. |  |
| genetic reagent  (*D. melanogaster*) | KC_gd_d1; MB419B | <https://doi.org/10.7554/eLife.04577.047> | MB419B | For further details on driver lines see Table S1. Available via <https://www.janelia.org/split-GAL4>. |  |
| genetic reagent  (*D. melanogaster*) | KC_gd_d2; MB607 | <https://doi.org/10.7554/eLife.04577.047> | MB607B | For further details on driver lines see Table S1. Available via <https://www.janelia.org/split-GAL4>. |  |
| genetic reagent  (*D. melanogaster*) | Kdm2_d1 | BDSC | BL-30819 | For further details on driver lines see Table S1. |  |
| genetic reagent  (*D. melanogaster*) | L1_d1; SS00693 | [https://doi.org/10.1016/j.neuron.2013.05.024](https://doi.org/10.1016/j.neuron.2013.05.024) | SS00693 | For further details on driver lines see Table S1. Available via <https://www.janelia.org/split-GAL4>. |  |
| genetic reagent  (*D. melanogaster*) | L1_d2; SS00691 | This paper | SS00691 | For further details on driver lines see Table S1. Available via <https://www.janelia.org/split-GAL4>. |  |
| genetic reagent  (*D. melanogaster*) | L1.L2_d1; SS00806 | [https://doi.org/10.1016/j.neuron.2013.05.024](https://doi.org/10.1016/j.neuron.2013.05.024) | SS00806 | For further details on driver lines see Table S1. Available via <https://www.janelia.org/split-GAL4>. |  |
| genetic reagent  (*D. melanogaster*) | L1.L2_d2; SS00797 | [https://doi.org/10.1016/j.neuron.2013.05.024](https://doi.org/10.1016/j.neuron.2013.05.024) | SS00797 | For further details on driver lines see Table S1. Available via <https://www.janelia.org/split-GAL4>. |  |
| genetic reagent  (*D. melanogaster*) | L2_d1; SS00801 | [https://doi.org/10.1016/j.neuron.2013.05.024](https://doi.org/10.1016/j.neuron.2013.05.024) | SS00801 | For further details on driver lines see Table S1. Available via <https://www.janelia.org/split-GAL4>. |  |
| genetic reagent  (*D. melanogaster*) | L3_d1; SS00696 | [https://doi.org/10.1016/j.neuron.2013.05.024](https://doi.org/10.1016/j.neuron.2013.05.024) | SS00696 | For further details on driver lines see Table S1. Available via <https://www.janelia.org/split-GAL4>. |  |
| genetic reagent  (*D. melanogaster*) | L4_d1; SS00789 | [https://doi.org/10.1016/j.neuron.2013.05.024](https://doi.org/10.1016/j.neuron.2013.05.024) | SS00789 | For further details on driver lines see Table S1. Available via <https://www.janelia.org/split-GAL4>. |  |
| genetic reagent  (*D. melanogaster*) | L5_d1; SS00692 | [https://doi.org/10.1016/j.neuron.2013.05.024](https://doi.org/10.1016/j.neuron.2013.05.024) | SS00692 | For further details on driver lines see Table S1. Available via <https://www.janelia.org/split-GAL4>. |  |
| genetic reagent  (*D. melanogaster*) | Lai_d1; SS55442 | This paper | SS55442 | For further details on driver lines see Table S1. Available via <https://www.janelia.org/split-GAL4>. |  |
| genetic reagent  (*D. melanogaster*) | Lat_d1; SS00657 | This paper | SS00657 | A gift from Heather Dionne, Rubin lab, Janelia Research Campus. For further details on driver lines see Table S1. Available via <https://www.janelia.org/split-GAL4>. |  |
| genetic reagent  (*D. melanogaster*) | Lawf1_d1; SS00800 | [https://doi.org/10.1016/j.neuron.2013.05.024](https://doi.org/10.1016/j.neuron.2013.05.024) | SS00800 | For further details on driver lines see Table S1. Available via <https://www.janelia.org/split-GAL4>. |  |
| genetic reagent  (*D. melanogaster*) | Lawf1_d2; SS00689 | This paper | SS00689 | For further details on driver lines see Table S1. Available via <https://www.janelia.org/split-GAL4>. |  |
| genetic reagent  (*D. melanogaster*) | Lawf2_d1; SS00698 | [https://doi.org/10.1016/j.neuron.2013.05.024](https://doi.org/10.1016/j.neuron.2013.05.024) | SS00698 | For further details on driver lines see Table S1. Available via <https://www.janelia.org/split-GAL4>. |  |
| genetic reagent  (*D. melanogaster*) | LC10a_d1; OL0019B | <https://doi.org/10.7554/eLife.21022.001> | OL0019B | For further details on driver lines see Table S1. Available via <https://www.janelia.org/split-GAL4>. |  |
| genetic reagent  (*D. melanogaster*) | LC10b_d1; SS03723 | <https://doi.org/10.7554/eLife.21022.001> | SS03723 | For further details on driver lines see Table S1. Available via <https://www.janelia.org/split-GAL4>. |  |
| genetic reagent  (*D. melanogaster*) | LC10bc_d1; SS00940 | <https://doi.org/10.7554/eLife.21022.001> | SS00940 | For further details on driver lines see Table S1. Available via <https://www.janelia.org/split-GAL4>. |  |
| genetic reagent  (*D. melanogaster*) | LC10d_d1; SS03822 | <https://doi.org/10.7554/eLife.21022.001> | SS03822 | For further details on driver lines see Table S1. Available via <https://www.janelia.org/split-GAL4>. |  |
| genetic reagent  (*D. melanogaster*) | LC16_d1; OL0046B | <https://doi.org/10.7554/eLife.21022.001> | OL0046B | For further details on driver lines see Table S1. Available via <https://www.janelia.org/split-GAL4>. |  |
| genetic reagent  (*D. melanogaster*) | LC16_d2; OL0092C | <https://doi.org/10.7554/eLife.21022.001> | OL0092C | For further details on driver lines see Table S1. Available via <https://www.janelia.org/split-GAL4>. |  |
| genetic reagent  (*D. melanogaster*) | LC4_d1; SS00766 | <https://doi.org/10.7554/eLife.21022.001> | SS00766 | For further details on driver lines see Table S1. Available via <https://www.janelia.org/split-GAL4>. |  |
| genetic reagent  (*D. melanogaster*) | LC6_d1; OL0070B | <https://doi.org/10.7554/eLife.21022.001> | OL0070B | For further details on driver lines see Table S1. Available via <https://www.janelia.org/split-GAL4>. |  |
| genetic reagent  (*D. melanogaster*) | LC6_d2; OL0218B | <https://doi.org/10.7554/eLife.21022.001> | OL0218B | For further details on driver lines see Table S1. Available via <https://www.janelia.org/split-GAL4>. |  |
| genetic reagent  (*D. melanogaster*) | lLNv_d1; SS00645 | This paper | SS00645 | A gift from Heather Dionne, Rubin lab, Janelia Research Campus. For further details on driver lines see Table S1. Available via <https://www.janelia.org/split-GAL4>. |  |
| genetic reagent  (*D. melanogaster*) | LLPC1_d1; SS02437 | This paper | SS02437 | For further details on driver lines see Table S1. Available via <https://www.janelia.org/split-GAL4>. |  |
| genetic reagent  (*D. melanogaster*) | LPC1_d1; SS02575 | This paper | SS02575 | For further details on driver lines see Table S1. Available via <https://www.janelia.org/split-GAL4>. |  |
| genetic reagent  (*D. melanogaster*) | LPC1_d2; SS02700 | This paper | SS02700 | For further details on driver lines see Table S1. Available via <https://www.janelia.org/split-GAL4>. |  |
| genetic reagent  (*D. melanogaster*) | LPi-34_d1; SS03656 | This paper | SS03656 | For further details on driver lines see Table S1. Available via <https://www.janelia.org/split-GAL4>. |  |
| genetic reagent  (*D. melanogaster*) | LPLC1_d1; OL0029B | <https://doi.org/10.7554/eLife.21022.001> | OL0029B | For further details on driver lines see Table S1. Available via <https://www.janelia.org/split-GAL4>. |  |
| genetic reagent  (*D. melanogaster*) | LPLC2_d1; OL0048B | <https://doi.org/10.7554/eLife.21022.001> | OL0048B | For further details on driver lines see Table S1. Available via <https://www.janelia.org/split-GAL4>. |  |
| genetic reagent  (*D. melanogaster*) | LPTC_HS.VS_d1; SS04438 | This paper | SS04438 | For further details on driver lines see Table S1. Available via <https://www.janelia.org/split-GAL4>. |  |
| genetic reagent  (*D. melanogaster*) | MBON_bp1_d1; MB057B | <https://doi.org/10.7554/eLife.04577.047> | MB057B | For further details on driver lines see Table S1. Available via <https://www.janelia.org/split-GAL4>. |  |
| genetic reagent  (*D. melanogaster*) | MBON_g1pedc_d1; MB112C | <https://doi.org/10.7554/eLife.04577.047> | MB112C | For further details on driver lines see Table S1. Available via <https://www.janelia.org/split-GAL4>. |  |
| genetic reagent  (*D. melanogaster*) | Mi1_d1; SS00809 | [https://doi.org/10.1016/j.neuron.2017.03.010](https://doi.org/10.1016/j.neuron.2017.03.010) | SS00809 | For further details on driver lines see Table S1. Available via <https://www.janelia.org/split-GAL4>. |  |
| genetic reagent  (*D. melanogaster*) | Mi15_d1; SS00321 | This paper | SS00321 | For further details on driver lines see Table S1. Available via <https://www.janelia.org/split-GAL4>. |  |
| genetic reagent  (*D. melanogaster*) | Mi15_d2; SS02425 | This paper | SS02425 | For further details on driver lines see Table S1. Available via <https://www.janelia.org/split-GAL4>. |  |
| genetic reagent  (*D. melanogaster*) | Mi4_d1; SS01019 | [https://doi.org/10.1016/j.neuron.2017.03.010](https://doi.org/10.1016/j.neuron.2017.03.010) | SS01019 | For further details on driver lines see Table S1. Available via <https://www.janelia.org/split-GAL4>. |  |
| genetic reagent  (*D. melanogaster*) | Mi9_d1; SS02432 | [https://doi.org/10.1016/j.neuron.2017.03.010](https://doi.org/10.1016/j.neuron.2017.03.010) | SS02432 | For further details on driver lines see Table S1. Available via <https://www.janelia.org/split-GAL4>. |  |
| genetic reagent  (*D. melanogaster*) | Mi9_d2; SS02448 | This paper | SS02448 | For further details on driver lines see Table S1. Available via <https://www.janelia.org/split-GAL4>. |  |
| genetic reagent  (*D. melanogaster*) | Muscles_App_d1 | BDSC | BL-1767 | For further details on driver lines see Table S1. |  |
| genetic reagent  (*D. melanogaster*) | Muscles_Head_d1 | BDSC | BL-1767 | For further details on driver lines see Table S1. |  |
| genetic reagent  (*D. melanogaster*) | Muscles_Head_d2 | BDSC | R10F02 | For further details on driver lines see Table S1. |  |
| genetic reagent  (*D. melanogaster*) | NPF_d1 | BDSC | BL-25681 | For further details on driver lines see Table S1. |  |
| genetic reagent  (*D. melanogaster*) | PAM_1_d1; MB109B | <https://doi.org/10.7554/eLife.04577.047> | MB109B | For further details on driver lines see Table S1. Available via <https://www.janelia.org/split-GAL4>. |  |
| genetic reagent  (*D. melanogaster*) | PAM_2_d1; MB196B | <https://doi.org/10.7554/eLife.04577.047> | MB196B | For further details on driver lines see Table S1. Available via <https://www.janelia.org/split-GAL4>. |  |
| genetic reagent  (*D. melanogaster*) | PAM_3_d1; MB213B | <https://doi.org/10.7554/eLife.04577.047> | MB213B | For further details on driver lines see Table S1. Available via <https://www.janelia.org/split-GAL4>. |  |
| genetic reagent  (*D. melanogaster*) | PAM_4_d1; MB312B | <https://doi.org/10.7554/eLife.04577.047> | MB312B | For further details on driver lines see Table S1. Available via <https://www.janelia.org/split-GAL4>. |  |
| genetic reagent  (*D. melanogaster*) | PAM_5_d1; MB315C | <https://doi.org/10.7554/eLife.04577.047> | MB315C | For further details on driver lines see Table S1. Available via <https://www.janelia.org/split-GAL4>. |  |
| genetic reagent  (*D. melanogaster*) | PAM_6_d1; MB042B | <https://doi.org/10.7554/eLife.04577.047> | MB042B | For further details on driver lines see Table S1. Available via <https://www.janelia.org/split-GAL4>. |  |
| genetic reagent  (*D. melanogaster*) | PAM_7_d1; MB043C | <https://doi.org/10.7554/eLife.04577.047> | MB043C | For further details on driver lines see Table S1. Available via <https://www.janelia.org/split-GAL4>. |  |
| genetic reagent  (*D. melanogaster*) | PAM_8_d1; MB441B | <https://doi.org/10.7554/eLife.04577.047> | MB441B | For further details on driver lines see Table S1. Available via <https://www.janelia.org/split-GAL4>. |  |
| genetic reagent  (*D. melanogaster*) | PAM_9_d1; MB063B | <https://doi.org/10.7554/eLife.04577.047> | MB063B | For further details on driver lines see Table S1. Available via <https://www.janelia.org/split-GAL4>. |  |
| genetic reagent  (*D. melanogaster*) | PB_1_d1; SS00116 | This paper | SS00116 | A gift from Tanya Wolff, Rubin lab, Janelia Research Campus. For further details on driver lines see Table S1. Available via <https://www.janelia.org/split-GAL4>. |  |
| genetic reagent  (*D. melanogaster*) | PB_2_d1; SS00090 | <https://doi.org/10.1002/cne.24512> | SS00090 | For further details on driver lines see Table S1. Available via <https://www.janelia.org/split-GAL4>. |  |
| genetic reagent  (*D. melanogaster*) | PB_3_d1; SS02268 | This paper | SS02268 | A gift from Tanya Wolff, Rubin lab, Janelia Research Campus. For further details on driver lines see Table S1. Available via <https://www.janelia.org/split-GAL4>. |  |
| genetic reagent  (*D. melanogaster*) | PB_4_d1; SS02204 | This paper | SS02204 | A gift from Tanya Wolff, Rubin lab, Janelia Research Campus. For further details on driver lines see Table S1. Available via <https://www.janelia.org/split-GAL4>. |  |
| genetic reagent  (*D. melanogaster*) | PB_4_d2; SS02302 | This paper | SS02302 | A gift from Tanya Wolff, Rubin lab, Janelia Research Campus. For further details on driver lines see Table S1. Available via <https://www.janelia.org/split-GAL4>. |  |
| genetic reagent  (*D. melanogaster*) | PB_5_d1; SS02255 | <https://doi.org/10.1002/cne.24512> | SS02255 | For further details on driver lines see Table S1. Available via <https://www.janelia.org/split-GAL4>. |  |
| genetic reagent  (*D. melanogaster*) | PB_6_d1; SS00078 | <https://doi.org/10.1002/cne.24512> | SS00078 | For further details on driver lines see Table S1. Available via <https://www.janelia.org/split-GAL4>. |  |
| genetic reagent  (*D. melanogaster*) | Pdf_d1 | BDSC | BL-6900 |  |  |
| genetic reagent  (*D. melanogaster*) | Pm3_d1; SS00328 | This paper | SS00328 | For further details on driver lines see Table S1. Available via <https://www.janelia.org/split-GAL4>. |  |
| genetic reagent  (*D. melanogaster*) | Pm4_d1; SS00317 | This paper | SS00317 | For further details on driver lines see Table S1. Available via <https://www.janelia.org/split-GAL4>. |  |
| genetic reagent  (*D. melanogaster*) | R1-6_d1; ninaEfl-GAL4 | This paper | ninaEfl-GAL4 | A gift from Barret Pfeiffer, Rubin lab. For further details on driver lines see Table S1. Available via <https://www.janelia.org/split-GAL4>. |  |
| genetic reagent  (*D. melanogaster*) | R7_d1 | BDSC | BL-8604 | For further details on driver lines see Table S1. Available via <https://www.janelia.org/split-GAL4>. |  |
| genetic reagent  (*D. melanogaster*) | R7_Rh3_d1 | BDSC | BL-7457 | For further details on driver lines see Table S1. |  |
| genetic reagent  (*D. melanogaster*) | R8_Rh5_d1 | BDSC | BL-7458 | For further details on driver lines see Table S1. |  |
| genetic reagent  (*D. melanogaster*) | R8_Rh6_d1 | BDSC | BL-7464 | For further details on driver lines see Table S1. |  |
| genetic reagent  (*D. melanogaster*) | T1_d1; SS00790 | This paper | SS00790 | For further details on driver lines see Table S1. Available via <https://www.janelia.org/split-GAL4>. |  |
| genetic reagent  (*D. melanogaster*) | T4_d1; SS02344 | This paper | SS02344 | For further details on driver lines see Table S1. Available via <https://www.janelia.org/split-GAL4>. |  |
| genetic reagent  (*D. melanogaster*) | T4_d2; SS23866 | This paper | SS23866 | For further details on driver lines see Table S1. Available via <https://www.janelia.org/split-GAL4>. |  |
| genetic reagent  (*D. melanogaster*) | T4.T5_d1; SS00324 | [https://doi.org/10.1016/j.neuron.2017.03.010](https://doi.org/10.1016/j.neuron.2017.03.010) | SS00324 | For further details on driver lines see Table S1. Available via <https://www.janelia.org/split-GAL4>. |  |
| genetic reagent  (*D. melanogaster*) | T4.T5_d2; SS21452 | This paper | SS21452 | For further details on driver lines see Table S1. Available via <https://www.janelia.org/split-GAL4>. |  |
| genetic reagent  (*D. melanogaster*) | T5_d1; SS25175 | This paper | SS25175 | For further details on driver lines see Table S1. Available via <https://www.janelia.org/split-GAL4>. |  |
| genetic reagent  (*D. melanogaster*) | T5_d2; SS23757 | This paper | SS23757 | For further details on driver lines see Table S1. Available via <https://www.janelia.org/split-GAL4>. |  |
| genetic reagent  (*D. melanogaster*) | Tm1_d1; SS00796 | This paper | SS00796 | For further details on driver lines see Table S1. Available via <https://www.janelia.org/split-GAL4>. |  |
| genetic reagent  (*D. melanogaster*) | Tm2_d1; SS00811 | This paper | SS00811 | For further details on driver lines see Table S1. Available via <https://www.janelia.org/split-GAL4>. |  |
| genetic reagent  (*D. melanogaster*) | Tm20_d1; SS00355 | This paper | SS00355 | For further details on driver lines see Table S1. Available via <https://www.janelia.org/split-GAL4>. |  |
| genetic reagent  (*D. melanogaster*) | Tm29_d1; SS00308 | This paper | SS00308 | For further details on driver lines see Table S1. Available via <https://www.janelia.org/split-GAL4>. |  |
| genetic reagent  (*D. melanogaster*) | Tm3_d1; SS00300 | [https://doi.org/10.1016/j.neuron.2017.03.010](https://doi.org/10.1016/j.neuron.2017.03.010) | SS00300 | For further details on driver lines see Table S1. Available via <https://www.janelia.org/split-GAL4>. |  |
| genetic reagent  (*D. melanogaster*) | Tm4_d1; SS00320 | This paper | SS00320 | For further details on driver lines see Table S1. Available via <https://www.janelia.org/split-GAL4>. |  |
| genetic reagent  (*D. melanogaster*) | Tm9_d1; SS00307 | This paper | SS00307 | For further details on driver lines see Table S1. Available via <https://www.janelia.org/split-GAL4>. |  |
| genetic reagent  (*D. melanogaster*) | TmY3_d1; SS00331 | This paper | SS00331 | For further details on driver lines see Table S1. Available via <https://www.janelia.org/split-GAL4>. |  |
| genetic reagent  (*D. melanogaster*) | TmY5a_d1; SS02594 | This paper | SS02594 | For further details on driver lines see Table S1. Available via <https://www.janelia.org/split-GAL4>. |  |
| genetic reagent  (*D. melanogaster*) | VGlut_d1; BL-60312 | BDSC | BL-60312 | For further details on driver lines see Table S1. Available via <https://www.janelia.org/split-GAL4>. |  |
| genetic reagent  (*D. melanogaster*) | UNC84-2XGFP | <https://doi.org/10.1093/nar/gks671> |  |  |  |
| genetic reagent  (*D. melanogaster*) | fkh-GFP;  PBac{y[+mDint2] w[+mC]=fkh-GFP.FPTB}VK00037 | BDSC | RRID:BDSC_43951 |  |  |
| genetic reagent  (*D. melanogaster*) | Ets65A-GFP; PBac{y[+mDint2] w[+mC]=Ets65A-GFP.FLAG}VK00037 | BDSC | RRID:BDSC_38640 |  |  |
| genetic reagent  (*D. melanogaster*) | Mi{PT-GFSTF.0}Nos[MI09718-GFSTF.0] | BDSC | RRID:BDSC_60278 |  |  |
| genetic reagent  (*D. melanogaster*) | Mi{Trojan-GAL4.1}Oamb[MI12417-TG4.1] | BDSC | RRID:BDSC_67506 |  |  |
| genetic reagent  (*D. melanogaster*) | Mi{Trojan-GAL4.1}Lim3[MI03817-TG4.1] | BDSC | RRID:BDSC_67450 |  |  |
| genetic reagent  (*D. melanogaster*) | Mi{PT-GFSTF.1}klg[MI02135-GFSTF.1] | BDSC | RRID:BDSC_59787 |  |  |
| genetic reagent  (*D. melanogaster*) | Mi{PT-GFSTF.2}GluClalpha[MI02890-GFSTF.2] | BDSC | RRID:BDSC_60533 |  |  |
| genetic reagent  (*D. melanogaster*) | Mi{PT-GFSTF.0}TfAP-2[MI04611-GFSTF.0] | BDSC | RRID:BDSC_61776 |  |  |
| genetic reagent  (*D. melanogaster*) | Mi{Trojan-GAL4.2}kn[MI15480-TG4.2 | BDSC | RRID:BDSC_67516 |  |  |
| genetic reagent  (*D. melanogaster*) | pJFRC12-10XUAS-IVS-myr::GFP in attP2 | BDSC | RRID:BDSC_32197 |  |  |
| genetic reagent  (*D. melanogaster*) | pJFRC19-13XLexAop2-IVS-myr::GFP in su(Hw)attP8 | BDSC | RRID:BDSC_32211 |  |  |
| genetic reagent  (*D. melanogaster*) | VAChT-FRT-STOP-FRT-HA; TI{TI}VAChT[FRT-STOP-FRT.HA] | BDSC | RRID:BDSC_76021 |  |  |
| genetic reagent  (*D. melanogaster*) | sens-FLP; ““w[*] P{y[+t7.7] w[+mC]=sens-FLPG5.C}attP18; wg[Sp-1]/CyO; sens[Ly-1]/TM6B, Tb[1]”” | BDSC | RRID:BDSC_55768 |  |  |
| genetic reagent  (*D. melanogaster*) | ey3.5FLP; ””P{w[+mC]=ey3.5-FLP.B}1, y[1] w[*]; CyO/In(2LR)Gla, wg[Gla-1] PPO1[Bc]”” | BDSC | RRID:BDSC_35542 |  |  |
| genetic reagent  (*D. melanogaster*) | pJFRC51-3XUAS-IVS-Syt::smHA in su(Hw)attP1, pJFRC225-5XUAS-IVS-myr::smFLAG in VK00005 | https://doi.org/10.1073/pnas.1506763112 |  |  |  |
| genetic reagent  (*D. melanogaster*) | MCFO-1; ””pBPhsFlp2::PEST in attP3; ;pJFRC201-10XUAS-FRT>STOP>FRT-myr::smGFP-HA in VK0005, pJFRC240-10X-UAS-FRT>STOP>FRT-myr::smGFP-V5-THS-10XUAS-FRT>STOP>FRT-myr::smGFP-FLAG in su(Hw)attP1”” | https://doi.org/10.1073/pnas.1506763112 | RRID:BDSC_64085 |  |  |
| genetic reagent  (*D. melanogaster*) | MCFO-7; ””R57C10-Flp2::PEST in in attP18; pJFRC210-10XUAS-FRT>STOP>FRT-myr::smGFP-OLLAS in attP2, pJFRC201-10XUAS-FRT>STOP>FRT-myr::smGFP-HA in VK0005, pJFRC240-10X-UAS-FRT>STOP>FRT-myr::smGFP-V5-THS-10XUAS-FRT>STOP>FRT-myr::smGFP-FLAG in su(Hw)attP1) ”” | https://doi.org/10.1073/pnas.1506763112 | RRID:BDSC_64091 |  |  |
| genetic reagent  (*D. melanogaster*) | 20XUAS-CsChrimson-mVenus in attP18 | https://doi.org/10.1038/nmeth.2836 | RRID:BDSC_55134 |  |  |
| antibody | anti-GFP rabbit polyclonal | ThermoFisher: A-11122 | RRID:AB_221569 | (1:1000) |  |
| antibody | anti-GFP mouse monoclonal 3E6 | ThermoFisher: A-11120 | RRID:AB_221568 | (1:100) |  |
| antibody | anti-dsRed rabbit polyclonal | Clontech Laboratories, Inc.: 632496 | RRID:AB_10013483 | (1:1000) |  |
| antibody | anti-HA rabbit monoclonal C29F4 | Cell Signaling Technologies: 3724S | RRID:AB_1549585 | (1:300) |  |
| antibody | anti-FLAG rat monoclonal DYKDDDDK Epitope Tag Antibody [L5], | Novus Biologicals: NBP1-06712 | RRID:AB_1625981 | (1:200) |  |
| antibody | DyLight 550 conjugated anti-V5 mouse monoclonal | AbD Serotec: MCA1360D550GA | RRID:AB_2687576 | (1:500) |  |
| antibody | DyLight 549 conjugated anti-V5 mouse monoclonal | AbD Serotec: MCA1360D549GA | RRID:AB_10850329 | (1:500) |  |
| antibody | anti-cockroach allatostatin (Ast7) mouse monoclonal 5F10 | Developmental Studies Hybridoma Bank (DSHB) | RRID:AB_528076 | (1:5) |  |
| antibody | anti-CadN rat monoclonal DN-Ex #8 | DSHB | RRID:AB_528121 | (1:20) |  |
| antibody | anti-chaoptin mouse monoclonal 24B10 | DSHB | RRID:AB_528161 | (1:20) |  |
| antibody | anti-Brp mouse monoclonal nc82 | DSHB | RRID:AB_2314866 | (1:30) |  |
| antibody | Anti-GFP antibody | ThermoFisher | G10362 RRID:AB_2536526 |  |  |
| Chemical compound | 1X Complete protease inhibitor | Sigma Aldrich | 5056489001 |  |  |
| Recombinant protein | 50mg/ml UltraPure BSA | ThermoFisher | AM2618 |  |  |
| Other | 10mg/ml Torula RNA | ThermoFisher | AM7118 |  |  |
| Other | Carboxyl-coated Dynabeads | ThermoFisher | 14306D |  |  |
| Recombinant proteins | Protein G Dynabeads | ThermoFisher | 10004D |  |  |
| Recombinant proteins | Protein A Dynabeads | ThermoFisher | 10002D |  |  |
|  | UNOsphere SUPra resin | Biorad | 1560218 |  |  |
| Recombinant protein | RNAsin | Promega | N2515 |  |  |
| Recombinant protein | IdeZ protease | NEB | P0770S |  |  |
| Recombinant protein | DNAseI | NEB | M0303L |  |  |
| Commercial assay or kit | ERCC standard spike-in RNA | ThermoFisher | 4456740 |  |  |
| Recombinant protein | Superasin | ThermoFisher | AM2696 |  |  |
| Chemical compound | Optiprep | Sigma Aldrich | D1556 |  |  |
| Other | 1 micron cup filter | Pluriselect | 43-50001-03 |  |  |
| Other | 10 micron cup filter | Sysmex | 04-0042-2314 |  |  |
| Other | 20 micron cup filter | Sysmex | 04-0042-2315 |  |  |
| Software, algorithms | seqtk (2012 Oct 16) | <https://github.com/lh3/seqtk> |  |  |  |
| Software, algorithms | kallisto 0.43.1 | <https://pachterlab.github.io/kallisto/> |  |  |  |
| Software, algorithms | STAR 2.5.3c | <https://github.com/alexdobin/STAR> |  |  |  |
| Software, algorithms | picard 1.9.1 | <http://broadinstitute.github.io/picard/> |  |  |  |
| Software, algorithms | R v3.3.1 | <https://www.r-project.org/> |  |  |  |
| Software, algorithms | RStan/Stan | <http://mc-stan.org/users/interfaces/rstan> |  |  |  |
